# A novel biomarker in the diagnosis of parapneumonic effusion: neutrophil gelatinase-associated lipocalin

**DOI:** 10.1186/2049-6958-9-49

**Published:** 2014-09-15

**Authors:** Aziz Gümüs, Sevket Ozkaya, Songul Ozyurt, Halit Cınarka, Aynur Kirbas, Unal Sahin, Ferah Ece

**Affiliations:** Department of Pulmonary Medicine, Faculty of Medicine, Recep Tayyip Erdogan University, Rize, Turkey; Department of Pulmonary Medicine, Faculty of Medicine, Bahcesehir University, Istanbul, Turkey; Department of Biochemistry, Faculty of Medicine, Recep Tayyip Erdogan University, Rize, Turkey

**Keywords:** Neutrophil gelatinase-associated lipocalin, NGAL, Pleural effusion, Parapneumonic effusion

## Abstract

**Background:**

The protein neutrophil gelatinase-associated lipocalin (NGAL) is a mediator synthesized and released by neutrophils. Its physiological function is as yet unclear. Levels in blood increase in several inflammatory diseases. High serum values indicate poor prognosis for several diseases. Pleural effusion may appear as the result of various pathologies. The most common cause is heart failure (HF). Other common causes include parapneumonic (PPE) and malignant (MPE) pleural effusions, and pulmonary embolism. Tubercular effusion (TE) is commonly encountered in Turkey and similar developing countries. The purpose of this study was to investigate the effectiveness of NGAL, a current inflammation marker, in discriminating between different etiological diseases that cause pleural effusion.

**Methods:**

The study was performed at the Recep Tayyip Erdoğan University Faculty of Medicine Chest Diseases Clinic. One hundred patients were included in the study, 25 with parapneumonic effusion, 25 with heart failure-related effusion, 25 with tubercular effusion and 25 with cancer-related effusion. NGAL was measured in patients’ serum and pleural fluids.

**Results:**

Serum NGAL levels in PPE (171 ± 56 ng/ml) were significantly higher (p < 0.001) than those in HF (86 ± 31 ng/ml), CA (103 ± 42 ng/ml) and TE (63 ± 19 ng/ml). Pleural NGAL levels were also significantly higher in PPE compared to HF, MPE and TE (p < 0.001). Serum NGAL levels exhibited a positive correlation with white blood cell (WBC), neutrophil, C-reactive protein (CRP), sedimentation, serum LDH, creatinine, pleural leukocyte and pleural neutrophil numbers. The most significant correlation was between NGAL level and WBC (p < 0.001, r = 0.579). Both serum and pleural NGAL levels are highly effective in differentiating patients with PPE from those without PPE (AUC: 0.910 and 0.790, respectively).

**Conclusions:**

NGAL can be used in the diagnosis of diseases with an acute inflammatory course. Serum and pleural NGAL levels can differentiate PPE from other diseases causing pleural fluid with high sensitivity and specificity.

## Background

Neutrophil gelatinase-associated lipocalin (NGAL), also known as lipocalin-2, is a 25 kDa protein [1] synthesized, stored and released under different clinical conditions in neutrophil granules [2]. It is released in the lung, colon, pancreas, breast and several healthy tissues. The function of this substance is still unclear, although it is thought to play a role in the body’s immune response. An increase in serum or plasma NGAL levels has been shown in acute and chronic inflammatory diseases, ischemic diseases such as stroke and myocardial infarction, metabolic diseases such as obesity and type 2 diabetes mellitus, acute or chronic kidney failure, following heart and kidney transplantation and in solid tumors such as lung, colon and breast cancer [3–5]. It is thought to be involved in the emergence and progression of several diseases [6]. In clinical practice it is used as a biomarker showing early acute renal injury [7].

Pleural effusion may appear as the result of various pathologies. The most common cause is heart failure (HF). Other common causes include parapneumonic (PPE) and malignant (MPE) pleural effusions, and pulmonary embolism. Tubercular effusion (TE) is commonly encountered in Turkey and similar developing countries. Diagnosis of the disease causing pleural effusion is possible after systematic evaluation and various interventional procedures. In pleural effusion of unknown origin, transudate and exudate must first be differentiated using thoracentesis. Light’s criteria described in 1972 are still used for this purpose [8]. Biomarkers capable of establishing etiological diagnosis without invasive procedures have been investigated in exudate fluids. An adenosine deaminase (ADA) level above 70 U/ml is regarded as adequate for diagnosing pleural tuberculosis and has eliminated the need for invasive techniques such as pleural biopsy [9].

Our scan of the literature revealed no studies involving NGAL measurement in serum and pleural fluid in the diagnosis of diseases leading to pleural effusion. The purpose of this study was to investigate the efficacy of the inflammation marker NGAL in differentiating various etiological diseases leading to pleural effusion.

## Methods

### Patients

The study was performed at the RecepTayyipErdoğan University Faculty of Medicine Chest Diseases Clinic, Turkey. Local ethical committee approval was obtained before the study commenced, and signed consent forms were received from patients. Patients presenting to the clinic or admitted to the department with pleural effusion between March, 2013, and May, 2014, were included. Total protein, albumin and lactic dehydrogenase (LDH) measurements were performed in serum and pleural fluid, and transudate/exudate differentiation was made on the basis of light criteria. One hundred patients aged over 18 and diagnosed with HF, PPE, TE or CA-related pleural effusion following advanced tests were enrolled. NGAL, total protein, albumin, LDH, total cholesterol and hemogram measurements were performed for all participants. Creatinine, CRP and sedimentation were investigated in blood only.

### Exclusion criteria

Subjects with chronic renal failure, receiving diuretic therapy due to heart failure or using antibiotics were excluded.

### Pleural and serum NGAL measurement

10 ml venous blood and 10 ml pleural fluid specimens were collected and centrifuged within 1 h (at 3000 g/min for 10 min). That part of the pleural fluid that was not precipitated and the blood serum were placed into Eppendorf tubes by pipette. Specimens were stored in a deep freeze at -80 degrees. NGAL measurements were performed when a sufficient number had been obtained. Measurements were performed quantitatively using the enzyme-linked immune absorbent assay (ELISA) method with Biovendor Research and Diagnostic Products (Heidelberg, Germany) NGAL antibodies.

### Statistical analysis

Statistical analyses were performed using SPSS (SPSS version 16; SPSS Inc., Chicago, IL, USA) software. Constant variables were expressed as mean ± SD and categorical variables as %. Student’s t test was used in the comparison of parametric variables and the Mann-Whitney U test in the comparison of non-parametric variables between two groups. For comparisons among more than two groups, ANOVA was used for parametric variables and the Kruskal-Wallis test for non-parametric variables. Correlations between variables were investigated using Spearman’s correlation analysis. The χ square test was used to compare categoric variables. ROC curve analysis was used to determine the specificity, sensitivity and cut-off value of NGAL in serum and pleural fluid to distinguish between transude and exudate. Significance was set at p < 0.05.

## Results

One hundred patients, 25 with heart failure-related pleural effusion (25%), 25 with cancer-related pleural effusion (25%), 25 with parapneumonic pleural effusion (25%) and 25 with tubercular pleural effusion (25%) were included in the study. Out of the 25 cancer cases, 21 were lung cancers (17 non-small cell lung cancer, 4 small cell lung cancer), 3 breast cancers and 1 non-Hodgkin’s lymphoma. Mean age of the patients in the TE group was significantly lower than that in the other groups (p < 0.001). There was no difference among groups in terms of gender. The inflammation markers serum leukocyte, sedimentation rate and CRP levels were significantly higher in the PPE group compared to the other groups (p < 0.001). A comparison of patients divided into etiological groups is shown in Table 1. Serum NGAL levels were significantly higher in the PPE group compared to the HF, MPE and TE groups. Mean NGAL values in these groups were 171 ± 56 ng/ml, 86 ± 31 ng/ml, 103 ± 42 ng/ml and 63 ± 19 ng/ml, respectively (p < 0.001). Serum NGAL levels in the MPE group were also higher than those in the TE group (p = 0.013). There were no other significant differences among the groups. Similarly, pleural effusion NGAL levels in the PPE group were significantly higher than those in the HF, MPE and TE groups. Mean NGAL values in these groups were 147 ± 71 ng/ml, 84 ± 23 ng/ml, 74 ± 22 ng/ml and 75 ± 43 ng/ml, respectively (p < 0.001). No differences in terms of pleural effusion NGAL levels were observed among the HF, MPE and TE groups.

**Table 1:** **Comparison of groups by etiology**

Causes of effusion (N)	HF (25)	MPE (25)	PPE (25)	TE (25)	p
Age*	75 ± 9	71 ± 12	69 ± 16	39 ± 12	<0.001
Sex (W/M)	4/21	7/18	5/50	11/14	0.154
NGAL(S) (ng/ml)**	86 ± 31	103 ± 42	171 ± 56	63 ± 19	<0.001
NGAL(Pl) (ng/ml)**	84 ± 23	74 ± 22	147 ± 71	75 ± 43	<0.001
NGAL rate (Pl/S)*	1,05 ± 0,35	0,86 ± 0,43	0,99 ± 0,72	1,27 ± 0,71	0.158
Sedimentation rate (mm/hour)*	32 ± 20	57 ± 18	72 ± 24	49 ± 11	<0.001
CRP (mg/dl)**	2,2 ± 1,7	5,6 ± 3,1	17,3 ± 7,9	10,6 ± 5,2	<0.001
WBC (/ml)**	6615 ± 1490	9967 ± 4234	13826 ± 4723	6911 ± 1101	<0.001
Neutrophils (/ml)**	5005 ± 2164	7202 ± 4854	11326 ± 5430	4802 ± 1273	<0.001
Pl. Leukocytes (/ml)**	571 ± 382	868 ± 1262	2841 ± 4302	3072 ± 1625	0.001
Pl. Neutrophils (/ml)**	149 ± 213	257 ± 466	2530 ± 3553	744 ± 406	<0.001

* = Variables with parametric distribution. ** = Variables with non-parametric distribution.

CRP, C-Reactive protein; HF, Heart failure; N, Number of patients; ng/ml, nanogram/milliliter; M, Men; MPE, Malignant pleural effusion; Pl, Pleural effusion; PPE, Parapneumonic pleural effusion; S, Serum; TE, Tubercular effusion; W, Women; WBC, White blood count.

**Figure 1:**
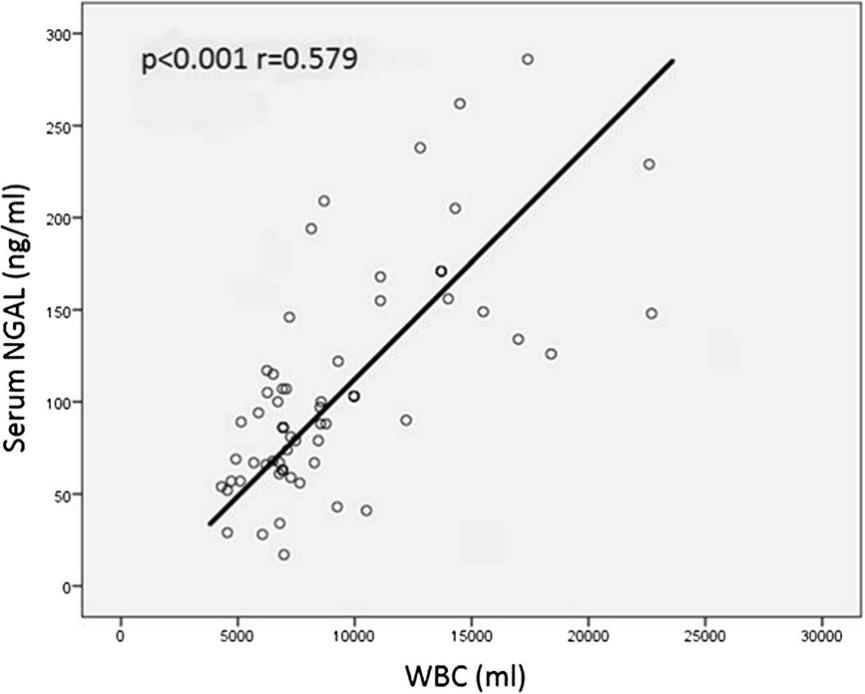
**Positive correlation between serum NGAL level and WBC.**

**Figure 2:**
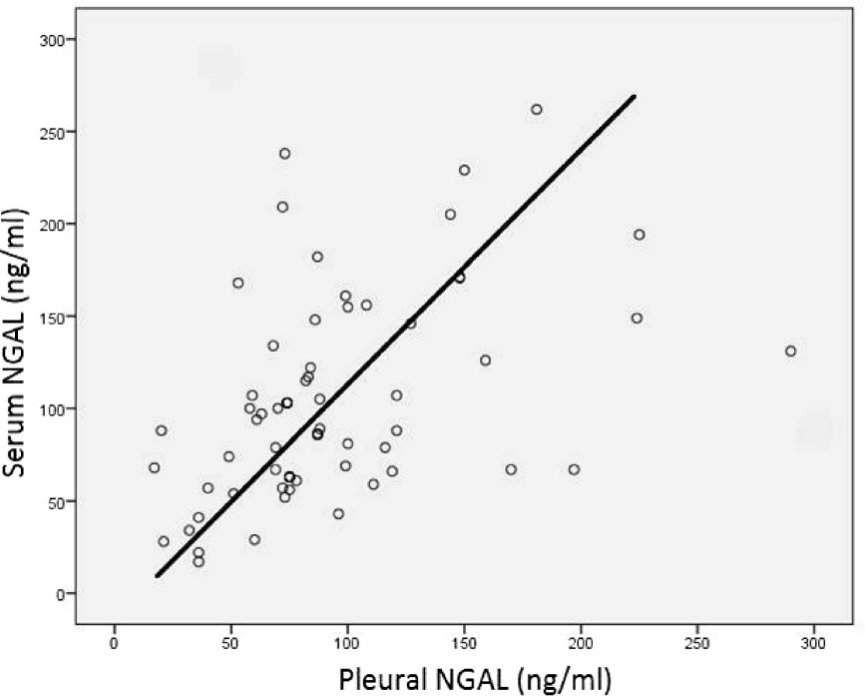
**Positive correlation between serum and pleural NGAL.**

**Figure 3:**
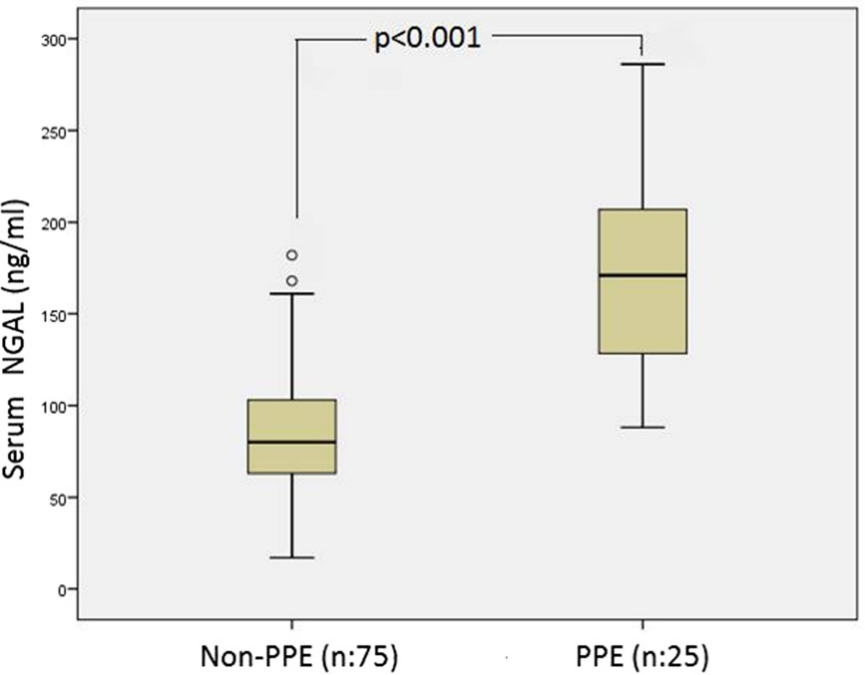
**Boxplot chart of serum NGAL levels of the PPE and non-PPE groups.**

**Figure 4:**
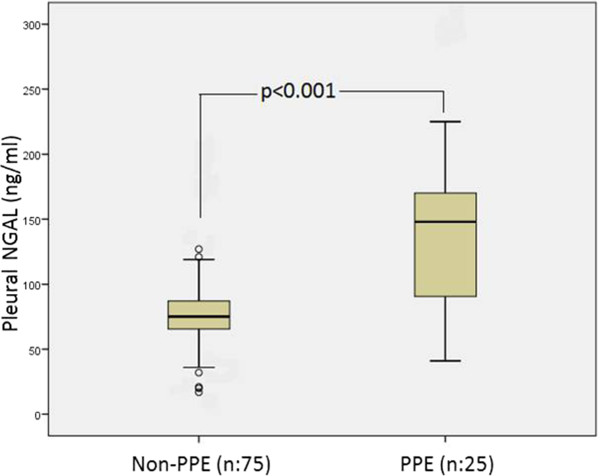
**Boxplot chart of pleural NGAL levels of the PPE and non-PPE groups.**

**Figure 5:**
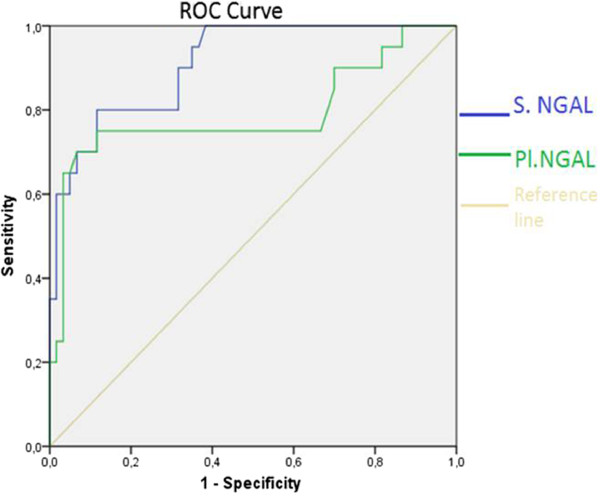
**ROC curve showing effectiveness of serum and Pleural NGAL in differentiating PPE.**

Spearman’s correlation analysis was performed in order to determine relations between serum and pleural NGAL levels and age and other constant variables. Serum NGAL levels were positively correlated with blood WBC, blood neutrophil numbers, CRP, sedimentation rate, serum LDH, creatinine, pleural WBC and pleural neutrophil numbers. Pleural NGAL was positively correlated with CRP, blood WBC, blood neutrophil numbers and serum LDH. However, there was no correlation between pleural NGAL and pleural WBC or pleural neutrophils (Table 2). The most significant correlation was between serum NGAL level and blood WBC (p < 0.001, r = 0.579). This correlation is shown in Figure 1. A significant positive correlation was found between serum NGAL and blood neutrophil number (p < 0.001, r = 0.504). Pleural NGAL rose in line with serum NGAL (p = 0.001, r = 0.366) (Figure 2).Since both serum and pleural NGAL levels were significantly elevated in the PPE group, the HF, MPE and TE group patients were combined into one non-PPE group. This group consisting of 75 non-PPE individuals was compared with the 25 PPE patients. Serum NGAL level in the non-PPE group was 84 ± 35 ng/ml, compared to 171 ± 51 ng/ml in the PPE group (p < 0.001). Similarly, pleural NGAL levels in the PPE group (148 ± 65 ng/ml) were significantly higher (p < 0.001) than in the non-PEE group (78 ± 30 ng/ml). The variation between the groups is shown in Figures 3 and 4. ROC curve analysis was performed to determine the efficacy of serum and pleural NGAL levels in discriminating between PPE and non-PPE patients. Both serum and pleural NGAL levels had high efficacy in discriminating between PPE and non-PPE patients. AUC for serum NGAL was 0.910, and AUC for pleural NGAL was 0.790. This characteristic of serum and pleural NGAL is shown in Figure 5. PPE patients could be differentiated from non-PPE patients with 89% specificity and 80% sensitivity at a cut-off value 124 ng/ml for serum NGAL, and with 89% specificity and 75% specificity at a cut-off value of 104 ng/ml for pleural NGAL. At a cut-off value of 188 ng/ml serum NGAL diagnosed PPE with 100% specificity. Pleural NGAL values of 210 ng/ml and above can differentiate PPE from other diseases with 100% specificity.

**Table 2:** **Correlation between serum and pleural fluid NGAL levels and constant variables**

	Serum NGAL		Pleural fluid NGAL	
	Spearman’s correlation coefficient (r=)	p	Spearman’s correlation coefficient (r=)	p
Age	0.109	0.337	0.093	0.410
CRP	0.473	<0.001	0.293	0.008
WBC	0.579	<0.001	0.445	<0.001
Neutrophils	0.504	<0.001	0.433	0.001
Sedimentation rate	0.347	0.002	0.107	0.347
LDH	0.457	<0.001	0.278	0.038
Creatinine	0.220	0.050	0.080	0.480
Pleural leukocytes	0.286	0.036	0.075	0.589
Pleural neutrophils	0.471	0.003	0.089	0.596
Pleural NGAL	0.366	0.001

## Discussion

Pleural effusion is one of the main causes of admissions to chest disease clinics. Clinical problems of pleural fluid origin represent approximately 4% of all applications to internal diseases clinics [10]. The first procedure to be performed when pleural fluid of unknown causes is encountered is thoracentesis and transudate/exudate differentiation. The exudate requires advanced examination. Several biomarkers have been investigated for the diagnosis of the primary disease without requiring invasive procedures. However, only pleural adenosine deaminase (ADA) measurement has entered into routine practice. ADA values above 70 U/ml are regarded as sufficient for diagnosis of pleural tuberculosis. ADA above 40 U/ml is regarded as sufficient for diagnosis in patients aged under 35 in regions in which tuberculosis is widespread [11].

In this study we investigated the ability of NGAL to identify specific diseases responsible for pleural effusion. We showed that both serum and pleural NGAL levels differentiated PPE from other causes of PPE, such as HF, MPE and tuberculosis. Serum NGAL levels established this differentiation more efficiently than pleural NGAL levels. Serum NGAL levels above 188 ng/ml and pleural NGAL levels above 210 ng/ml can distinguish PPE with a 100% specificity. The original feature of this study is that it is the first one measuring NGAL in pleural fluid. Several previous studies have measured NGAL in blood serum or plasma and less frequently in urine. Most studies concerning NGAL have been concerned with it as an early marker of acute renal injury [12, 13]. NGAL is specifically released in response to nephron damage in acute renal injury, and its rise in blood and urine is a poor prognostic factor [14, 15]. NGAL measurements directed toward acute renal injury have gradually begun appearing in routine clinical practice. NGAL has rarely been investigated in body fluids other than blood and urine. NGAL measurement has been performed in phlegm in chronic obstructive pulmonary disease [16, 17] and in bronchoalveolar lavage [18]. In our study, serum and pleural NGAL were high in PPE and pneumonia, that is an acute infection. Previous studies have shown that NGAL rises in acute infections. Axelsson et al. reported that serum NGAL levels in acute peritonitis were 10 times higher than those of a healthy control group [19]. Chakraborty et al. reported that serum NGAL levels were correlated with the severity of the disease in patients with acute pancreatitis [4]. One recent study involving children with pneumonia in Africa showed that serum NGAL levels were highly specific and sensitive in differentiating severe pneumonia and bacterial pneumonia [20]. That same study reported that at a cut-off value of ≥ 130.1 ng/ml for NGAL was able to predict bacterial pneumonia with 85.7% specificity and 83.7% sensitivity. Another recent study reported that plasma NGAL levels increased significantly in patients with disseminated intravascular coagulation [21]. In our study, serum NGAL levels were increased in acute infections, and both serum and pleural NGAL levels in patients with PPE (an acute, infectious complication of pneumonia) were significantly higher than those in patients with heart failure, cancer and tuberculosis. At a cut-off value of 124 ng/ml, NGAL was able to differentiate PPE from the other diseases at the high levels of 89% specificity and 80% sensitivity. A cut-off value of 104 ng/ml for pleural NGAL predicted patients with PPE with 89% specificity and 75% sensitivity. PPE can be diagnosed in patients with difficult to diagnose pleural effusion if serum NGAL level is ≥ 188 and/or pleural NGAL level is ≥ 210. Of course, these data need to be confirmed in wider series studies.

Another important finding in this study is the positive correlation between serum NGAL level and the acute inflammation markers WBC, neutrophils and CRP. In a study of patients with HIV, Landro et al. reported a positive correlation between serum NGAL and neutrophil numbers [22]. Similarly, Allegra et al. showed a positive correlation between serum NGAL levels and leukocyte (WBC) and neutrophil numbers [23]. Our results are in close agreement with those of these studies. Serum NGAL levels rise significantly in acute inflammation, and increase together with those of other inflammation markers. NGAL would seem to be a biomarker that can be used in the diagnosis or monitoring of acute infection. We think that wider-ranging studies are needed to elucidate this property of NGAL. Eagan et al. [24] determined a positive correlation between serum NGAL levels and neutrophil, CRP and creatinine levels in patients with chronic obstructive pulmonary disease (COPD). NGAL rises in correlation with other inflammation markers, not only in acute but also in chronic inflammation. A novel biomarker has been reported in order to discriminate parapneumonic from other exudative effusions. High concentrations of Pentraxin-3(PTX-3) in pleural effusions are very sensitive to differentiate PPE from non-PPE, while they do not seem able to differentiate between uncomplicated and complicated pleural effusions [25].

Last but not least, this study has revealed that pleural/serum NGAL levels were similar in all four disease groups. NGAL levels do not seem practicable in the differentiation between transudate and exudate and specific diseases.

## Conclusions

In conclusion, we can say this is the first study in which NGAL has been investigated in terms of diagnosis in diseases progressing with pleural effusion. Both serum NGAL and pleural NGAL measurements can differentiate PPE from pleural effusion associated with heart failure, cancer and tuberculosis. In addition, NGAL can be used in the diagnosis of PPE and pneumonia.

## References

[1] Kjeldsen L, Johnsen AH, Sengeløv H, Borregaard N (1993). Isolation and primary structure of NGAL, a novel protein associated with human neutrophil gelatinase. J Biol Chem.

[2] Kjeldsen L, Cowland JB, Borregaard N (2000). Human neutrophil gelatinase-associated lipocalin and homologous proteins in rat and mouse. Biochim Biophys Acta.

[3] Fjaertoft G, Foucard T, Xu S, Venge P (2005). Human neutrophil lipocalin (HNL) as a diagnostic tool in children with acute infections: a study of the kinetics. Acta Paediatr.

[4] Chakraborty S, Kaur S, Muddana V, Sharma N, Wittel UA, Papachristou GI, Whitcomb D, Brand RE, Batra SK (2010). Elevated serum neutrophil gelatinase-associated lipocalinis an early predictor of severity and outcome in acute pancreatitis. Am J Gastroenterol.

[5] Yndestad A, Landro L, Ueland T, Dahl CP, Flo TH, Vinge LE, Espevik T, Froland SS, Husberg C, Christensen G, Dickstein K, Kjekshus J, Oie E, Gullestad L, Aukrust P (2009). Increased systemic and myocardial expression of neutrophil gelatinase-associated lipocalin in clinical and experimental heart failure. Eur Heart J.

[6] Chakraborty S, Kaur S, Guha S, Batra SK (2012). The multifaceted roles of neutrophil gelatinase associated lipocalin (NGAL) in inflammation and cancer. Biochim Biophys Acta.

[7] Devarajan P (2008). Neutrophil gelatinase-associated lipocalin (NGAL): a new marker of kidney disease. Scand J Clin Lab Invest Suppl.

[8] Light RW (1997). Diagnostic principles in pleural disease. Eur Respir J.

[9] Metintaş M, Ozlü T, Metintaş M, Karadağ M, Kaya A (2010). Plevralsıvılıhastanındeğerlendirilmesi. Solunum Sistemi Hastalıkları.

[10] Light RW (2007). Pleural Diseases.

[11] (2012). Global Tuberculosis Control-WHO Report 2012.

[12] Singer E, Markó L, Paragas N, Barasch J, Dragun D, Müller DN, Budde K, Schmidt-Ott KM (2013). Neutrophil gelatinase-associated lipocalin: pathophysiology and clinical applications. Acta Physiol (Oxf).

[13] Hjortrup PB, Haase N, Wetterslev M, Perner A (2013). Clinical review: predictive value of neutrophil gelatinase-associated lipocalin for acute kidney injury in intensive care patients. Crit Care.

[14] Liu KD, Yang W, Anderson AH, Feldman HI, Demirjian S, Hamano T, He J, Lash J, Lustigova E, Rosas SE, Simonson MS, Tao K, Hsu CY (2013). Chronic Renal Insufficiency Cohort (CRIC) study investigators. Urine neutrophil gelatinase-associated lipocalin levels do not improve risk prediction of progressive chronic kidney disease. Kidney Int.

[15] Kai K, Yamaguchi T, Yoshimatsu Y, Kinoshita J, Teranishi M, Takasaki W (2013). Neutrophil gelatinase-associated lipocalin, a sensitive urinary biomarker of acute kidney injury in dogs receiving gentamicin. J Toxicol Sci.

[16] Iwamoto H, Gao J, Koskela J, Kinnula V, Kobayashi H, Laitinen T, Mazur W (2014). Differences in plasma and sputum biomarkers between COPD and COPD-asthma overlap. Eur Respir J.

[17] Keatings VM, Barnes PJ (1997). Granulocyte activation markers in induced sputum: comparison between chronic obstructive pulmonary disease, asthma, and normal subjects. Am J Respir Crit Care Med.

[18] Finlay GA, Russell KJ, McMahon KJ, D'arcy EM, Masterson JB, FitzGerald MX, O'Connor CM (1997). Elevated levels of matrix metalloproteinases in bronchoalveolar lavage fluid of emphysematous patients. Thorax.

[19] Axelsson L, Bergenfeldt M, Ohlsson K (1995). Studies of the release and turnover of a human neutrophil lipocalin. Scand J Clin Lab Invest.

[20] Huang H, Ideh RC, Gitau E, Thézénas ML, Jallow M, Ebruke B, Chimah O, Oluwalana C, Karanja H, Mackenzie G, Adegbola RA, Kwiatkowski D, Kessler BM, Berkley JA, Howie SR, Casals-Pascual C (2014). Discovery and validation of biomarkers to guide clinical management of pneumonia in african children. Clin Infect Dis.

[21] In JW, Kim JE, Jeong JS, Song SH, Kim HK (2014). Diagnostic and prognostic significance of neutrophil gelatinase-associated lipocalin in disseminated intravascular coagulation. Clin Chim Acta.

[22] Landro L, Damås JK, Flo TH, Heggelund L, Ueland T, Tjonnfjord GE, Espevik T, Aukrust P, Froland SS (2008). Decreased serum lipocalin-2 levels in human immunodeficiency virus-infected patients: increase during highly active anti-retroviral therapy. Clin Exp Immunol.

[23] Allegra A, Alonci A, Bellomo G, Campo S, Cannavò A, Penna G, Russo S, Centorrino R, Gerace D, Petrungaro A, Musolino C (2011). Increased serum levels of neutrophil gelatinase-associated lipocalin in patients with essential thrombocythemia and polycythemia vera. Leuk Lymphoma.

[24] Eagan TM, Damås JK, Ueland T, Voll-Aanerud M, Mollnes TE, Hardie JA, Bakke PS, Aukrust P (2010). Neutrophil gelatinase-associated lipocalin: a biomarker in COPD. Chest.

[25] Ozsu S, Abul Y, Mentese A, Bektas H, Uzun A, Ozlu T, Porcel JM (2013). Pentraxin-3: A novel biomarker for discriminating parapneumonic from other exudative effusions. Respirology.

